# Parallel genome-scale loss of function screens in 216 cancer cell lines for the identification of context-specific genetic dependencies

**DOI:** 10.1038/sdata.2014.35

**Published:** 2014-09-30

**Authors:** Glenn S Cowley, Barbara A Weir, Francisca Vazquez, Pablo Tamayo, Justine A Scott, Scott Rusin, Alexandra East-Seletsky, Levi D Ali, William FJ Gerath, Sarah E Pantel, Patrick H Lizotte, Guozhi Jiang, Jessica Hsiao, Aviad Tsherniak, Elizabeth Dwinell, Simon Aoyama, Michael Okamoto, William Harrington, Ellen Gelfand, Thomas M Green, Mark J Tomko, Shuba Gopal, Terence C Wong, Hubo Li, Sara Howell, Nicolas Stransky, Ted Liefeld, Dongkeun Jang, Jonathan Bistline, Barbara Hill Meyers, Scott A Armstrong, Ken C Anderson, Kimberly Stegmaier, Michael Reich, David Pellman, Jesse S Boehm, Jill P Mesirov, Todd R Golub, David E Root, William C Hahn

**Affiliations:** 1 Broad Institute of Harvard and MIT, 7 Cambridge Center, Cambridge, Massachusetts 02142, USA; 2 Department of Medical, Dana-Farber Cancer Institute, 450 Brookline Avenue, Boston, Massachusetts 02215, USA; 3 Department of Pediatric Oncology, Dana-Farber Cancer Institute, 450 Brookline Avenue, Boston, Massachusetts 02215, USA; 4 Center for Cancer Genome Discovery, Dana-Farber Cancer Institute, 450 Brookline Avenue, Boston, Massachusetts 02215, USA; 5 Departments of Medicine, Brigham and Women’s Hospital, Harvard Medical School, Boston, Massachusetts 02115, USA; 6 Blueprint Medicines, Inc. 215 1st Street, Cambridge, Massachusetts 02142, USA; 7 Memorial Sloan Kettering, 1275 York Ave, New York, New York 10065, USA

## Abstract

Using a genome-scale, lentivirally delivered shRNA library, we performed massively parallel pooled shRNA screens in 216 cancer cell lines to identify genes that are required for cell proliferation and/or viability. Cell line dependencies on 11,000 genes were interrogated by 5 shRNAs per gene. The proliferation effect of each shRNA in each cell line was assessed by transducing a population of 11M cells with one shRNA-virus per cell and determining the relative enrichment or depletion of each of the 54,000 shRNAs after 16 population doublings using Next Generation Sequencing. All the cell lines were screened using standardized conditions to best assess differential genetic dependencies across cell lines. When combined with genomic characterization of these cell lines, this dataset facilitates the linkage of genetic dependencies with specific cellular contexts (e.g., gene mutations or cell lineage). To enable such comparisons, we developed and provided a bioinformatics tool to identify linear and nonlinear correlations between these features.

## Background & Summary

Genome characterization efforts describe an increasingly comprehensive list of genetic alterations that occur in human cancers, but their contributions to the proliferation or survival of cancers often remains obscure. Furthermore, we lack a systematic understanding of the genetic vulnerabilities of human cancer cells as a function of these genetic alterations and the context in which they occur. A complementary effort to systematically assess the genetic requirements of cancer cells in many cellular contexts will help to decipher the roles of specific mutations and the vulnerabilities that these genetic alterations induce. The identification of these context-specific cancer-cell vulnerabilities is the motivation for the generation of the data set described here.

To identify genes that have a context-specific effect on cell viability, we performed short hairpin RNA (shRNA) screens in a large number of cell lines in a highly parallel manner, in an effort named Project Achilles. Although screens to identify proliferation/survival genes have been performed in individual cells, Project Achilles data sets are unique in the number of genes and cell lines screened. Genome-wide parallel pooled screens has been performed with 12 (ref. 1), 70 (ref. 2) and 102 (ref. 3) cell lines of a variety of lineages. The data set described here, Achilles 2.4, extends and advances these previously released data sets by increasing the number of cell lines that have been screened and employing a different deconvolution method using next generation sequencing that yields a wider dynamic range and more quantitative assessment than earlier-generation deconvolution methods.

**Figure 1:**
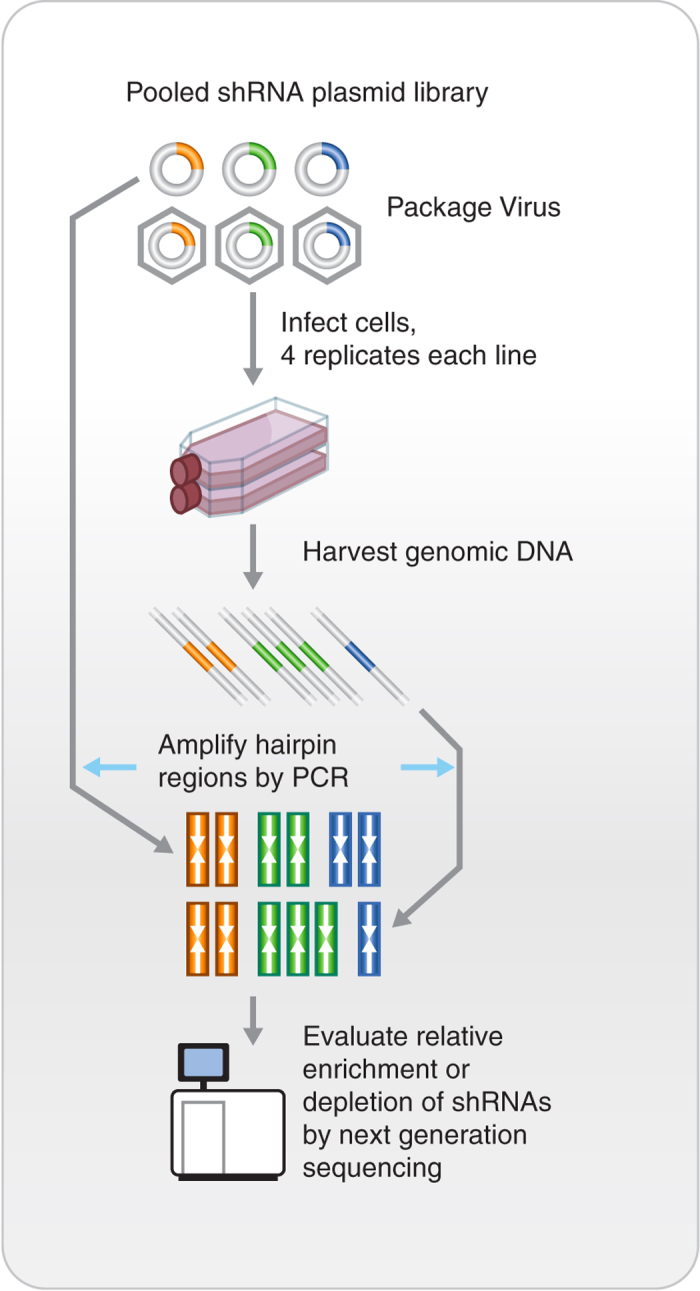
Schematic representation of the schema used for pooled shRNA screening.

The screening pipeline used to create this data set has been previously described^3^. Briefly, a pool of 54,020 shRNA plasmids targeting ~11,000 genes was used to infect the cell lines with a minimum representation of 200 cells per shRNA in each of 4 replicates. Cells were then propagated for 16 population doublings or 40 days in culture, whichever came first. The relative levels of the shRNA plasmids represented in the cell-pool gDNA were measured using Illumina-based sequencing and compared to the initial plasmid pool (Figure 1).

The Project Achilles data set facilitates the discovery of context-specific dependencies, genes that when suppressed only have an effect on the viability of the particular cell lines, combined with the discovery of biomarkers associated with these differential sensitivities. Using Project Achilles 2.0 (ref. 3) and Project Achilles 2.4, the ‘oncogene addictions’ of many known oncogenes such as PIK3CA, KRAS, and BRAF are identified by showing that cell lines harboring mutations of these genes exhibit higher sensitivity to their suppression. Furthermore, presence of these oncogenes in cells predicts dependency on other genes, for example *PIK3CA* ^mut^ cell lines have preferential dependency on MTOR. In addition to these known dependencies, we recently identified *SMARCA2* and *ARID1B* as essential genes in cell lines with *SMARCA4* and *ARID1A* mutations, respectively^4,5^.

In addition to these single-gene based relationships, the Achilles data set can be exploited to discover pathways to which specific tumor subtypes are preferentially dependent. One example of using such approach is the discovery of the dependency of WNT active tumors on members of the YAP pathway^6^. By making the data and analytical tools available to the scientific community, we expect that a number of new vulnerabilities will be revealed.

We made Achilles 2.4 available to the scientific community in the Project Achilles portal (www.broadinstitute.org/achilles). In addition to the shRNA level data, a gene-level dataset generated using the ATARiS algorithm^7^ is also available for download. ATARiS combines data from the multiple distinct shRNAs per target gene across many cell lines to reduce the contribution of off-target effects while reinforcing the on-target effects. Moreover, specific genes can be queried and the dependency profile for such genes across cell lines can be individually downloaded from the portal. To facilitate the discovery of molecular and cellular correlates of the dependencies profiles, we created an analytical tool that we named PARIS (Probability Analysis by Ranked Information Score) and made it available to the scientific community in GenePattern (www.genepattern.org).

## Methods

### Cell line information

The majority of cell lines (179) were obtained from the Cancer Cell Line Encyclopedia (www.broadinstitute.org/ccle). Cell line information, including source is listed in ’Table1_screening_information.xls’ (Data Citation 1). Tumor type and growth media conditions, also used for screening, were obtained from the CCLE project. For cell lines not obtained from CCLE, media conditions used by the source laboratory were employed. Cell doubling time is calculated from the lentivirally infected cells during the course of the experiment. Days in culture, calculated from the date of infection until the date of the harvest, and passage number, based on the number of splits that occurred during the time in culture, refer to the time point of the sample that was used for data collection specific to each cell line.

### SNP fingerprinting

To ensure the identity of the cell lines and rule out the possibility of cross-contamination during the screening process, a SNP fingerprinting quality control step was implemented. SNP fingerprinting matches a panel of reference SNP genotypes for a cell line, with genotypes assayed after the screening process. The reference set of SNP genotypes used for most samples was derived from the Affymetrix SNP6.0 array birdseed genotypes from the Cancer Cell Line Encyclopedia project^8,9^. Cell lines not present in CCLE were genotyped for the same panel of SNPs with either the Sequenom or Fluidigm platform and/or also profiled with SNP6.0 arrays before screening.

Two genotyping platforms, Sequenom and Fluidigm, were used to assay different panels of SNPs, which overlapped those present on the Affymetrix SNP6.0 array. DNA was isolated as described in the screening methods (below).

Briefly the Sequenom protocol is as follows: SNPs are amplified in PCR reactions that contain a maximum of 24 loci. The Single Base Extension reaction is then performed on the Shrimp Alkaline Phosphatase treated PCR product using iPLEX-GOLD enzyme and mass-modified terminators (Sequenom, SanDiego). A small volume of reaction is then loaded onto each position of a 384-well SpectroCHIP preloaded with matrix (3-hydroxypicolinic acid). SpectroCHIPs are analyzed in automated mode by a MassArray MALDI-TOF Compact system with a solid phase laser mass spectrometer (Bruker Daltonics Inc., 2005). The resulting spectra are called by real-time SpectroCaller algorithm and analyzed by MassArray Typer v.4.0 software which combines base caller with the clustering algorithm.

For Fluidigm Fingerprinting, we utilize 4 HX Fluidigm IFC chip loaders and 4 FC1 cyclers for the 96.96 dynamic array. To support this process, we use an Agilent Bravo and the BioMark HD system for the liquid handling steps of the process.

### Screening

We previously performed a genome-wide pooled shRNA screening of 102 cancer cell lines in quadruplicate (Achilles v2.0) to identify essential genes^3^. Using a library of 54,020 shRNAs targeting 11,194 genes individual shRNAs were lentivirally delivered to the cells. The abundance of the shRNAs was measured after the cells were propagated for 16 populations doublings or 40 days in culture, whichever came first, and compared to the initial DNA plasmid pool. To generate Achilles v2.4 we used the genomic DNA from these 102 cell lines and re-measured their abundance using a next generation sequencing approach. Four lines (A2780, F5, NCI-H82 and OVMANA) that were screened previously failed our new QC guidelines implemented with the new sequencing deconvolution pipeline (see below). An additional 143 cell lines were screened using a similar protocol. In total, high quality data from 216 cell lines make up the final Achilles v2.4 dataset. The media conditions used for all cell lines are listed in ‘Table1_screening_information.xls’ (Data Citation 1).

### Deconvolution of pooled screening by NGS

Deconvolution was performed similar to that described in Ashton *et al*.^10^. Briefly, the shRNA region was PCR amplified from the purified gDNA using the following conditions: 5 μl primary PCR primer mix, 4 μl dNTP mix, 1x Ex Taq buffer, 0.75 μl of Ex TaqDNA polymerase (Takara), and up to 10 μg genomic DNA in a total reaction volume of 100 μl. A total of 140 μg gDNA was used as template from each replicate. Thermal cycler PCR conditions consisted of heating samples to 95 °C for 5 min; 15 cycles of 94 °C for 30 s, 65 °C for 30 s, and 72 °C for 20 s; and 72 °C for 5 min. PCR reactions were then pooled per sample. A secondary PCR step was performed containing 5 μM of common barcoded 3′ primer, 8 μl dNTP mix, 1x Ex Taq buffer, 1.5 μLEx TaqDNA polymerase, and 30 μl of the primary PCR mix for a total volume of 90 μl. 10 μl of independent 5′ barcoded primers are then added into each reaction, after which the 100 μl total volume is divided into two 50 μl final reactions. Thermal cycler conditions for secondary PCR are as follows: 95 °C for 5 min; 15 cycles of 94 °C for 30 s, 58 °C for 30 s, and 72 °C for 20 s; and 72 °C for 5 min. Individual 50 μl reactions are then re-pooled. Reactions are then run on a 2% agarose gel and intensity-normalized. Equal amounts of samples, based on gel intensity are then mixed and gel-purified using a 2% agarose gel. Samples were sequence using a custom sequencing primer using standard Illumina conditions.

Primary PCR Primers:

5′: AATGGACTATCATATGCTTACCGTAACTTGAAAGTATTTCG

3′: CTTTAGTTTGTATGTCTGTTGCTATTATGTCTACTATTCTTTCCC

Secondary PCR Primers:

5′(BC):AATGATACGGCGACCACCGAGAAAGTATTTCGATTTCTTGGCTTTATATATCTTGTGGANNNNACGA

3′: CAAGCAGAAGACGGCATACGAGCTCTTCCGATCTTGTGGATGAATACTGCCATTTGTCTC

Custom Sequencing primer:

GAGAAAGTATTTCGATTTCTTGGCTTTATATATCTTGTGGA

For current methods please visit: http://www.broadinstitute.org/rnai/public/resources/protocols

20 replicates were multiplexed into a single Ilumina sample, and run on multiple lanes to achieve a minimum of 1^7^reads per replicate.

### Data processing pipeline

#### Read count normalization

Raw 45-mer reads were extracted from fastq files and binned into reads containing each unique 4-mer PCR primer barcode used for each screening replicate. Within each replicate, instances of the 21-mer shRNA sequence was counted, using the TRC reference list of all 21-mer hairpin sequences expected. This generated a matrix of the counts of raw Illumina reads for each shRNA in each screening replicate. These counts were normalized to the total number of reads collected for each replicate to account for the (modestly) variable read depth of each replicate. This was performed using the following equation:

Normalized shRNA value=log_2_ [(Raw read value for shRNA)/(Total raw read value for Replicate) ×1e6] +1

A GenePattern module to perform both the extraction of raw reads counts and to convert into a normalized data file named PoolQ will be available soon in GenePattern (http://genepattern.org).

#### Sample quality control

Quality control for replicate cell line samples consisted of two measures: replicate reproducibility and a measure of the overall distribution of shRNA normalized and logged read counts. The Pearson correlation between all replicate samples was calculated and the 75th percentile of the correlation of all non-replicate pairs (0.6795) was chosen as the threshold used to fail individual replicate samples. A measure of the overall distribution of each replicate sample was scored by calculating its 75th percentile. Those individual replicate samples that had a score less than the mean—1 standard deviation of all scores were removed from further analysis. In addition, any cell line that lacked 3 replicates passing both of these QC metrics was also removed. The GenePattern module ‘ReplicatesQC’ was used to run these metrics and identified replicate samples to be removed. Four additional cell lines were removed from the final dataset because they were engineered cell lines.

#### Achilles data processing GenePattern pipeline

Normalized and log_2_ transformed read counts per replicate sample (Data Citation 1) were processed in a GenePattern pipeline. The pipeline consisted of individual GenePattern modules (Supplementary Figure 1 and available here: http://genepattern.org and in the GParc repository: http://www.broadinstitute.org/software/gparc/), each responsible for a specific processing step. During the period of Illumina data collection, changes in both the cluster kit used (cBot v7 for early samples to cBot v8 for later samples), and the SBS kit (SBS v2 for earlier samples and v3 for later samples) were implemented. To minimize any technical error due to the variation in Illumina chemistry, we kept the data for each subset of samples independent for those collected using the cBotV7/sbsv2 kits and those collected using the cBOTv8/sbsv3 kits. Separate files corresponding to samples processed under different sequencing chemistry and software conditions were maintained in the pipeline until the correct reference DNA pool sample was mapped to each sample. Briefly, the pipeline starts with modules that remove undesirable shRNAs and failing QC replicate samples. These modules are ‘FilterLowshRNAs’, which removes shRNAs that start at low abundance in the plasmid DNA pool (median of ≤1 logged normalized read counts), ‘shRNAremoveOverlap’, which removes one of a pair of shRNAs that have an offset of <3 basepairs and ‘removeSamples’, which removes a list of failing replicates. The pipeline then calculates fold change values per shRNA per replicate using the ‘shRNAfoldChange’ module and normalizes the shRNA depletion values per replicate cell line to the same scale, using quantile normalization with the ‘NormLines’ module. Remaining replicates of each cell line were then collapsed to a single value per cell line, per shRNA using the ‘shRNAcollapseReps’ module. The last step in the data processing pipeline maps shRNAs to gene symbols, using a mapping file ‘CP0004_20131120_19mer_trans_v1.chip’ (Data Citation 1) and the ‘shRNAmapGenes’ module. Multiple shRNAs can be mapped to the same genes in the final shRNA-level data file (Data Citation 1), depending on this transcriptome mapping.

## Data Records

Data files have been deposited in the FigShare data repository as one text-based README file, four.gct formatted files, 3 tables as either.xls or tab delimited text and one.chip mapping file (Data Citation 1).

File 1. ‘Achilles_Analysis_README_v2.4.3.txt’

This is a README file outlining the data processing steps that occur between the initial logged and normalized read count files and the final shRNA- and gene-levels files.

File 2. ‘cBOTv7_sbsv2_allreps_log.gct’

The logged and normalized read counts from the pooled screening of the first group of cancer cell lines, performed in quadruplicate, and the appropriate DNA reference. This is a.gct formatted file, with replicate cell lines in columns and shRNAs in rows. Sequences of shRNA barcodes are in the 1st column (labeled ‘Name’), with a blank 2nd column (labeled ‘Description’).

File 3. ‘cBOTv8_sbsv3_allreps_log.gct’

The logged and normalized read counts from the pooled screening of the second group of cancer cell lines, performed in quadruplicate, and the appropriate DNA reference. This is a.gct formatted file, with replicate cell lines in columns and shRNAs in rows. Sequences of shRNA barcodes are in the 1st column (labeled ‘Name’), with a blank 2nd column (labeled ‘Description’).

File 4. ‘Achilles_QC_v2.4.3.rnai.gct’

The final shRNA-level file obtained after processing formatted as a.gct file, with cell lines in columns and shRNAs in rows. Sequences of shRNA barcodes are in the 1st column (labeled ‘Name’) and gene names mapped to those shRNAs are in the 2nd column (labeled ‘Description’).

File 5. ‘Achilles_QC_v2.4.3.rnai.Gs.gct’

The final gene-level file obtained after analysis of the shRNA level file with the ATARiS algorithm. This is a.gct formatted file, with cell lines in columns and ATARiS gene solutions in rows. ATARiS gene solutions are in the 1st column (labeled ‘Name’) and gene names are in the 2nd column (labeled ‘Description’).

File 6. ‘Achilles_QC_v2.4.3.shRNA.table.txt

The shRNA quality file produced after analysis of the shRNA level file with the ATARiS algorithm. This is a tab-delimited text file, with shRNAs in rows. The columns contain annotation information for each shRNA, including consistency scores and whether each shRNA was used in the resulting gene solution.

File 7. ‘Table1_screening information.xls’

The file of cell line information, including annotations about screening conditions, are present in this.xls formatted file. Described in more detail in the Methods section.

File 8. ‘Table2_SNP_genotyping.xls’

The file of SNP genotypes, per cell line, are present in this.xls formatted file. Described in more detail in the Methods section.

File 9. ‘CP0004_20131120_19mer_trans_v1.chip’

The shRNA to gene mapping file. This is a tab delimited text file with shRNA barcode sequences in rows, along with the mapping of each to gene transcript identifier, gene identifier and gene symbol.

## Technical Validation

### Sequencing deconvolves pooled shRNA data accurately, as assayed by an artificial dilution series

**Figure 2:**
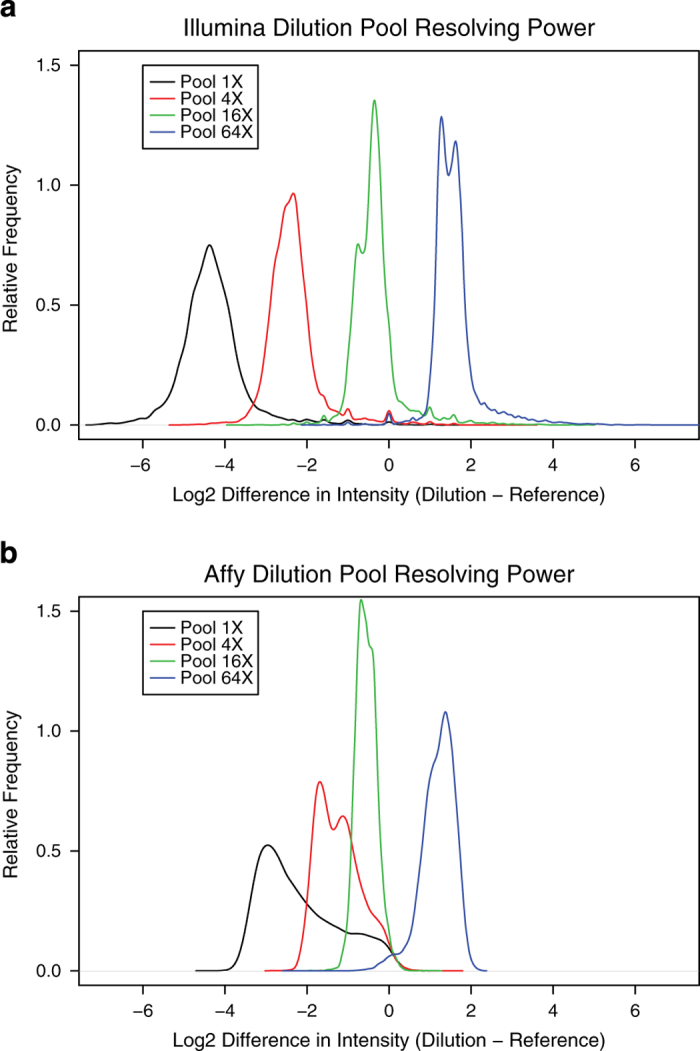
Assessment of data accuracy using DNA pools containing known relative proportions of DNA. Two 45,000-shRNA pools were created by combining 4 subsets of the shRNA library plasmids (labeled in black, red, green, and blue) in a 1:1:1:1 ratio of concentrations for the ‘Reference pool’ and in a 1:4:16:64 ratio for the ‘Dilution pool.’ The observed separation of the 4 subsets of shRNAs according to their known relative proportions in the 2 pools illustrates the ability of (**a**) NGS and (**b**) Affymetrix arrays to deconvolve the pooled shRNA library.

The performance of sequencing deconvolution was evaluated using engineered plasmid pools containing known relative proportions of DNA. Two 45,000-shRNA pools were created by combining 4 subsets of the shRNA library plasmids (labeled in black, red, green, blue, each consisting of ≈11,000 different plasmids) in a 1:1:1:1 ratio of concentration for the Reference pool and in a 1:4:16:64 ratio for the Dilution series pool. We show that 4-fold changes in relative shRNA abundance can be accurately shown by NGS (Illumina) sequencing, similar to that seen previously by custom Affymetrix arrays^1^ (Figure 2a,b).

### Accuracy of pooled screen measurements from sequencing deconvolution

**Figure 3:**
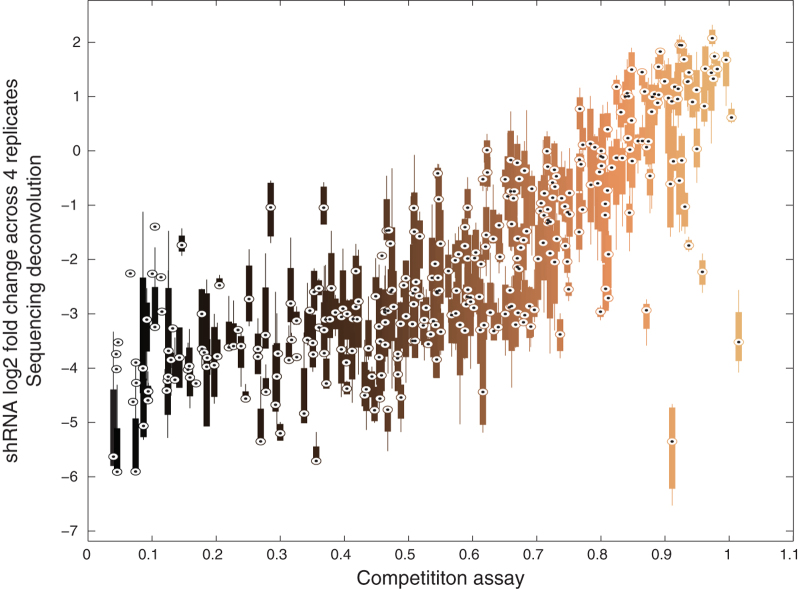
Comparison of pooled screen measurements from sequencing deconvolution against individual shRNA proliferation tests. The relative abundance (fold change values) of 350 shRNAs measured from sequencing deconvolution of four OVCAR-8 replicates (y-axis) are plotted against the relative abundance of OVCAR-8 cells (x-axis) infected with each shRNA encoded in a GFP+ plasmid, measured at 7 days post infection in the competition assay ^3^. The circled dot indicates the median value, boxes represent the 25th to 75th percentile and whiskers extend to the full range of the data for those 4 replicates.

We used a previously generated dataset of 350 shRNAs tested in competition assays in OVCAR-8 cells^3^ to compare with the values obtained using sequencing to deconvolve our pooled screening data. The percentage depletion of these shRNAs in the competition assay was correlated to their fold change in the pooled screening (Figure 3). The four replicates of OVCAR-8 had Spearman correlations to the competition assay that ranged between 0.75 and 0.77, indicating that sequencing deconvolution of our pooled screening provides an accurate measure of the effect of the shRNAs on cell viability.

### Dataset quality is enhanced by tracking and confirming cell line sample identity after screening

SNP fingerprinting was performed to validate the cell line identity after pooled screening. Birdseed genotypes from Affymetrix SNP6.0 arrays for 79 SNPs were primarily used as references for cell line identity and are listed by their dbSNP identifier in ‘Table 2_SNP_genotyping.xls’ (Data Citation 1), along with a designation of which fingerprinting platform was used for comparison. In some cases, a reference panel was genotyped before screening commenced. Fluidigm or Sequenom fingerprints after screening were extracted and compared to the reference, using the GenePattern module ‘FPmatching’ (http://genepattern.org). This table also contains information about the number of SNPs used for matching and the fraction matching between the reference and post-screen fingerprints. The ability to match post-screening results back to reference cell line genotypes confirms cell line identity in the screening results and ensures the correct use of previously collected genomic information from the CCLE project.

### Screening conditions do not lead to batch effects in the data

**Figure 4:**
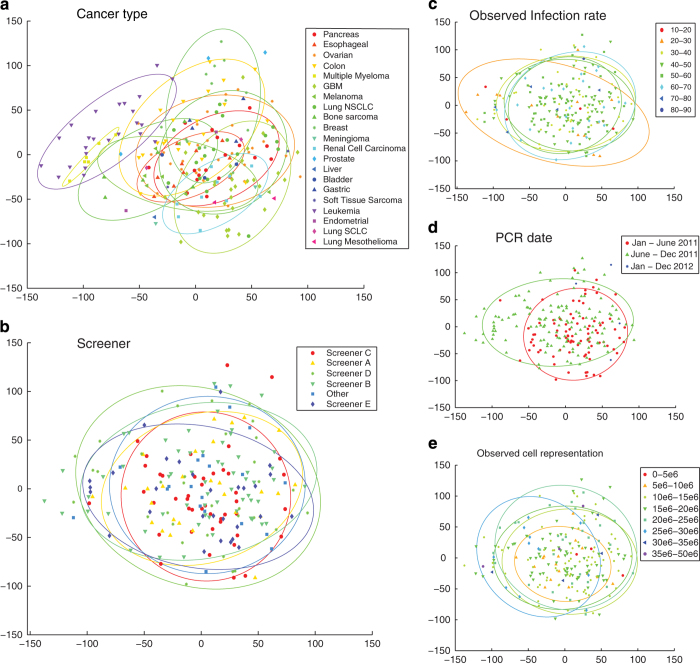
Evaluation of batch effect from differences in screening conditions. The first principal component (x-axis) was plotted against the second principal component (y-axis) using the shRNA-level data for all 216 cell lines. Each point is an individual cell line, and is colored by (**a**) cancer type, (**b**) screener, (**c**) observed infection rate of each screen, (**d**) date of the PCR reaction, and (**e**) observed cell representation of each screen. Ellipses are drawn around colored groups with greater than 5 examples, to aid in visualization.

Principal component analysis (PCA) was performed on the quantile-normalized shRNA level data to identify systematic variation among groups of cell lines. Scatterplots of the first two principal components (variables that account for the most variation in the data) show biological diversity between lineages, as seen in Figure 4a. This particularly differentiates the hematopoietic lineages like multiple myelomas and leukemias from solid malignancies. However, PCA also shows that screening conditions like infection rate, observed cell representation, date of PCR and the identity of the screener do not lead to batch effects (Figure 4b–e).

### Replicate cell line screens are reproducible

**Figure 5:**
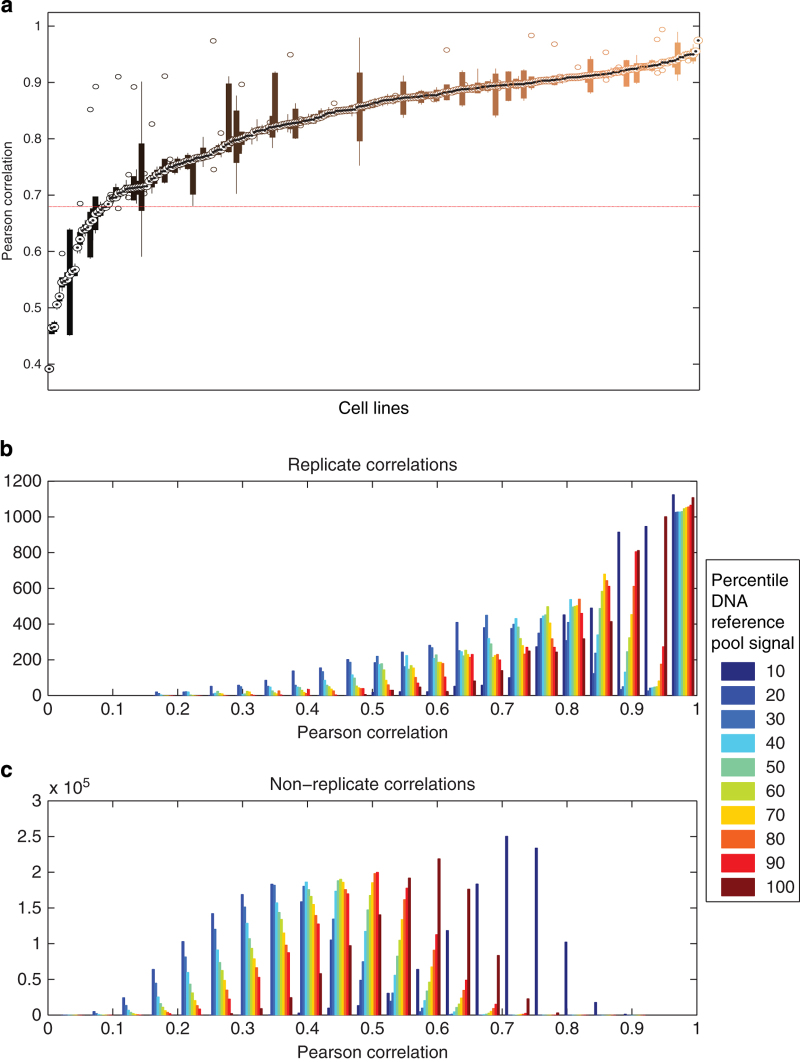
Assessment of reproducibility by measuring intra- and inter-replicate correlation. (**a**) A boxplot of correlation between replicates (y-axis) plotted for each cell line (x-axis) shows the range of replicate-replicate correlations. The circled dot indicates the median value, boxes represent the 25th to 75th percentile and whiskers extend to the full range of the data not considered outliers for each cell line. A line indicating the threshold for passing quality control is in red. Histograms of (**b**) all intra-replicate correlations and (**c**) all inter-replicate (non-replicate) correlations show overall that replicate correlations are higher than non-replicate correlations. Colors indicate the percentile of signal in the initial DNA reference pool.

As outlined in the sample quality control methods, the Pearson correlation within cell line replicates (intra-replicate) was calculated and compared with non-replicate pairs of samples (inter-replicate). The majority of cell lines have replicate correlations above the calculated quality control threshold (0.6795), highlighting the reproducibility of these screens (Figure 5a). In addition, when the shRNAs are divided into deciles based on the initial DNA reference pool signal, the intra-replicate correlations (Fig. 5b) of these shRNA groups are higher than their corresponding inter-replicate correlations (Fig. 5c). In general, the higher the initial DNA reference signal is, the higher both intra- and inter-replicate correlations are. One exception is the group of shRNAs within the lowest initial reference signal, as shRNAs that start out with a low signal can more easily drop out (have no signal) and look correlated in a loss of function screen.

## Usage Notes

### Project achilles portal

The Project Achilles Portal was developed at the Broad Institute to make the Achilles data sets more visible and easily available to the scientific community. In addition to the data set described here, our previously published data set^3^ is also hosted in this Portal. Project Achilles is an ongoing project and as additional data is generated, these data sets will be made available in the Portal when they become ready for public release. Moreover, we are continuously evaluating our data processing steps and re-iterations of the same data sets might be released in the Portal if they present a significant improvement.

The datasets are organized by version and with each version, data is available for download at the shRNA level and summarized at the gene-level using the ATARiS algorithm (http://www.broadinstitute.org/ataris/)^7^. We preferentially use the gene level scores in our downstream analysis since this takes into account the consistency of the different shRNA values for one gene across cell lines, and thus helps to maximize the on-target effects of shRNAs. In addition to the data at the shRNA and gene level, a sample information file, an shRNA to gene mapping file and a detailed description of the processing steps are provided which each version of the data. The data can be downloaded or launched using GENE-E (http://www.broadinstitute.org/cancer/software/GENE-E/).

The Portal also provides the users with the ability to search the data for specific genes. The gene page contains information on the shRNAs targeting such a gene present in the library, whether they contribute to the gene-summary score (ATARiS solution) and a consistency score (provides a score for each shRNA that represents the confidence that its observed phenotypic effects are the result of on-target gene suppression). A sortable heatmap with the shRNA and ATARiS scores per cell line is also shown.

### Data mining using PARIS (GenePattern module)

PARIS is a sensitive and general information-based feature selection method. A target profile of interest is identified, such as mutation status of an oncogene, and then the method selects the top *RNAi* essentiality profiles (shRNA- or gene-based) that best match the target profile in a collection of samples according to a rescaled normalized mutual information score (*RNMI*). As new metrics are evaluated, PARIS may be modified to support them.

The joint entropy *H(t, x)* and the Mutual Information, *MI(t, x)* between the target profile *t* and a given essentiality profile *x* are function of empirical probability distributions^11^

(1)H(t,x)=−∬P(t,x)logP(t,x)dtdx

(2)MI(t,x)=∬P(t,x)logP(t,x)P(t)P(x)dtdx

These probability distributions are determined from the data profiles using kernel density and cross-validation bandwidth estimation^12–14^. The mutual information is normalized (*NMI*) using the joint entropy,

(3)NMI(t,x)=MI(t,x)H(t,x).

This provides a universal metric^15^ that takes into account differences in entropy across essentiality profiles. We also rescale the *NMI* with the score of the target against itself,

(4)RNMI(t,x)=sign(ρ(t,x))NMI(t,x)NMI(t,t),

and add a ‘directionality’ factor according to the sign of the correlation coefficient ρ(t,x). In this way a perfect match (anti-match) corresponds to a score of +1 (−1) and a random match to 0. The significance of a given *RNMI* matching score is estimated by an empirical permutation test where the target values are randomly permuted and compared with all the essentiality profiles in order to make a global null distribution and compute nominal p-values and False Discovery Rates^16^. The *RNMI* matching score has important advantages when compared to other association metrics such as increased sensitivity to non-linear correlations and wider dynamic range at the top of the matching scale which is especially useful when comparing against multiple genomic features. The use of information-based measures of association is not new^17,18^ but it has only been applied systematically to practical inferential problems, and genomics in particular, over the last decade^19–23^. Recently there has been a renewed appreciation of the potential of information-based approaches^17,24,25^.

We made the PARIS publically available as a GenePattern module (http://genepattern.org) and a tutorial is available in the Project Achilles Portal. The Cancer Cell Line Encyclopedia portal (http://www.broadinstitute.org/ccle) is an excellent resource for obtaining genomic information on most of the Project Achilles-screened cell lines, to use in PARIS and other data mining tools.

## Additional information

**How to cite this article:** Cowley, G. S. *et al.* Parallel genome-scale loss of function screens in 216 cancer cell lines for the identification of context-specific genetic dependencies. *Sci. Data* 1:140035 doi: 10.1038/sdata.2014.35 (2014).

## Supplementary Material

Supplementary Information
