# Supplementary material for: Parallel genome-scale loss of function screens in 216 cancer cell lines for the identification of context-specific genetic dependencies
Source: Sci Data. 2014 Sep 30;1:140035. doi: 10.1038/sdata.2014.35 (PMC4432652; doi:10.1038/sdata.2014.35)
Supplement: Supplementary Information [file sdata201435-s1.pdf]

Data processing and analysis workflow

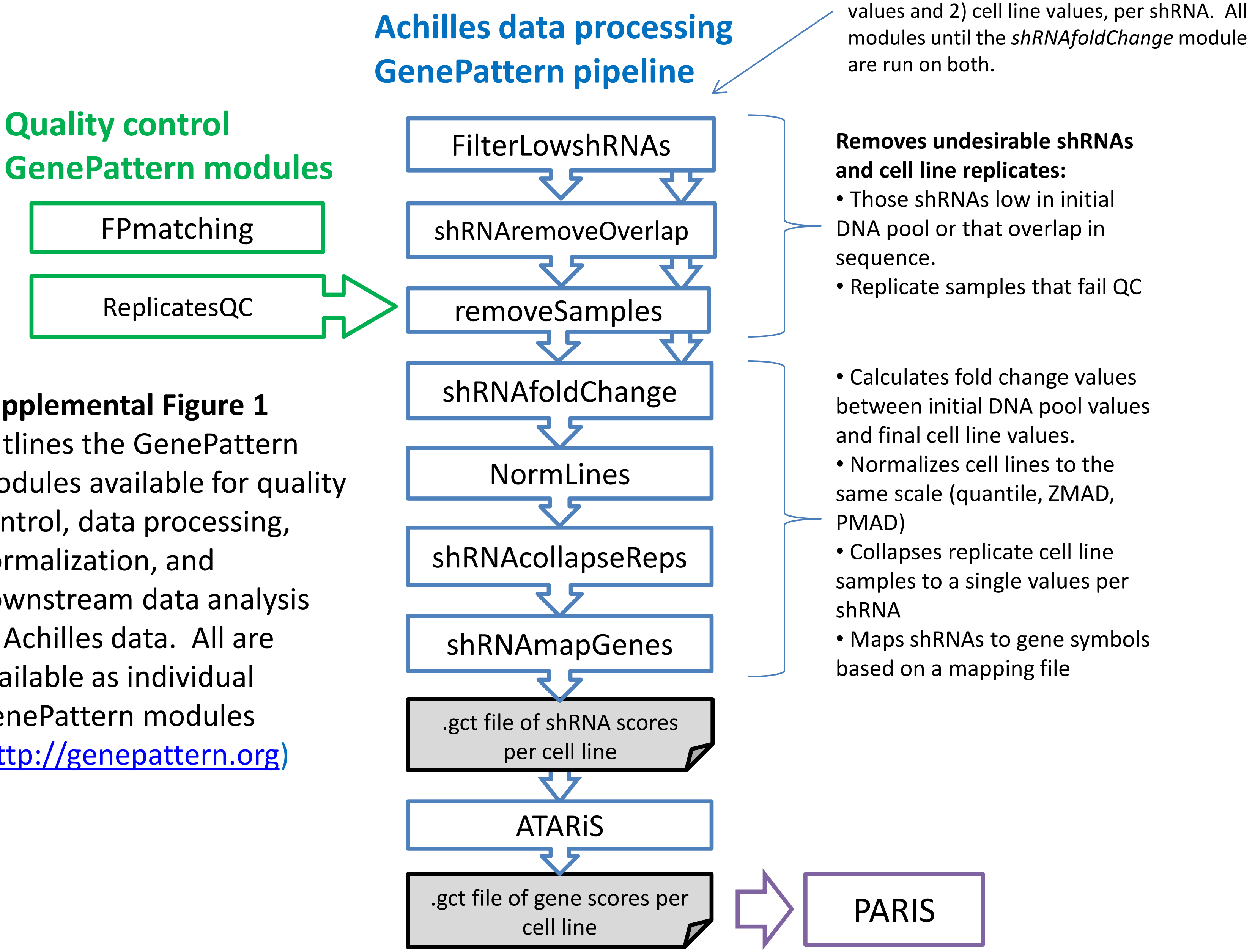

Supplemental Figure 1 outlines the GenePattern modules available for quality control, data processing, normalization, and downstream data analysis of Achilles data. All are available as individual GenePattern modules (http://genepattern.org)
